# Decellularization and recellularization of the ovary for bioengineering applications; studies in the mouse

**DOI:** 10.1186/s12958-020-00630-y

**Published:** 2020-07-23

**Authors:** Ahmed Baker Alshaikh, Arvind Manikantan Padma, Matilda Dehlin, Randa Akouri, Min Jong Song, Mats Brännström, Mats Hellström

**Affiliations:** 1 Laboratory for Transplantation and Regenerative Medicine, Sahlgrenska Academy, University of Gothenburg, Kvinnokliniken, Blå stråket 6, SE-413 45 Göteborg, Sweden; 2 Department of Obstetrics and Gynecology, Sahlgrenska Academy, University of Gothenburg, Gothenburg, Sweden; 3 Department of Obstetrics & Gynecology, Yeouido St. Mary’s Hospital, The Catholic University of Korea, Seoul, Republic of Korea; 4 Stockholm IVF-EUGIN, Stockholm, Sweden

**Keywords:** Ovary, Decellularization, Recellularization, Tissue engineering, Extracellular matrix

## Abstract

**Background:**

Fertility preservation is particularly challenging in young women diagnosed with hematopoietic cancers, as transplantation of cryopreserved ovarian cortex in these women carries the risk for re-introducing cancer cells. Therefore, the construction of a bioengineered ovary that can accommodate isolated small follicles was proposed as an alternative to minimize the risk of malignancy transmission. Various options for viable bioengineered scaffolds have been reported in the literature. Previously, we reported three protocols for producing mouse ovarian scaffolds with the decellularization technique. The present study examined these scaffolds further, specifically with regards to their extracellular composition, biocompatibility and ability to support recellularization with mesenchymal stem cells.

**Material and methods:**

Three decellularization protocols based on 0.5% sodium dodecyl sulfate (Protocol 1; P1), or 2% sodium deoxycholate (P2), or a combination of the two detergents (P3) were applied to produce three types of scaffolds. The levels of collagen, elastin and sulfated glycosaminoglycans (sGAGs) were quantified in the remaining extracellular matrix. Detailed immunofluorescence and scanning electron microscopy imaging were conducted to assess the morphology and recellularization efficiency of the constructs after 14 days in vitro utilizing red fluorescent protein-labelled mesenchymal stem cells.

**Results:**

All protocols efficiently removed the DNA while the elastin content was not significantly reduced during the procedures. The SDS-protocol (P1) reduced the sGAG and the collagen content more than the SDC-protocol (P2). All scaffolds were biocompatible and recellularization was successful, particularly in several P2-derived scaffolds. The cells were extensively distributed throughout the constructs, with a denser distribution observed towards the ovarian cortex. The cell density was not significantly different (400 to 550 cells/mm^2^) between scaffold types. However, there was a tendency towards a higher cell density in the SDC-derived constructs. Scanning electron microscope images showed fibrous scaffolds with a dense repopulated surface structure.

**Conclusions:**

While there were differences in the key structural macromolecules between protocols, all scaffolds were biocompatible and showed effective recellularization. The results indicate that our SDC-protocol might be better than our SDS-protocol. However, additional studies are necessary to determine their suitability for attachment of small follicles and folliculogenesis.

## Introduction

Radiotherapy and chemotherapy against cancer are commonly associated with reproductive disorders. These treatment-induced gonadotoxic effects may include a reduction in the number of primordial follicles, vascular damage and ovarian cortical fibrosis [1, 2]. Many treated women therefore experience premature ovarian insufficiency that is associated with early menopause and infertility. Improved anti-cancer regimes have significantly increased survival rates among cancer patients and it is therefore important to consider fertility after treatment and other quality-of-life factors. Options for fertility preservation in female cancer patients include ovarian/uterus transposition, embryo/oocyte vitrification or ovarian cortex cryopreservation [3–6]. In many cases, ovarian cortex transplantation can successfully restore fertility for female cancer survivors [7]. However, these methods are still considered unsafe for women diagnosed with hematopoietic cancers due to the risk of reintroducing cancer cells [8].

Therefore, in vitro preantral follicle stimulation and folliculogenesis have extensively been investigated with the aim to obtain viable oocytes suitable for in vitro fertilization [9, 10]. However, preantral follicles are challenging to culture without the natural supporting structure of the ovary that can conform to the remarkable follicular size-increase that occurs during folliculogenesis. A multi-step culture system was proposed as a solution for providing support during folliculogenesis in vitro [11]. Additionally, successful pre-clinical studies showed that biomaterials can be used as supporting structures for follicular growth. These scaffolds include fibrin- and/or alginate matrices, or three-dimensional (3D) printed cross-linked sharp-angled gelatin fibers and it has been demonstrated that viable oocytes can be aspirated from these constructs to eventually produce healthy offspring in rodents [12–16]. These positive findings have been confirmed by several independent labs and serve as proof-of-concept that a bioengineered ovary can support follicular growth in small mammals. However, the follicular growth distinctive to larger mammals, including humans, requires better biomaterials that are more compliant than currently existing scaffolds [17]. Alternatively, the use of sequential biomaterials with different composition may be required at different stages of folliculogenesis [18]. Biological scaffolds developed by a method called decellularization have been found to have several advantages in bioengineering applications in many systems, including female reproductive organs [19, 20]. These scaffolds are composed of a tissue-specific extracellular matrix (ECM) and were shown to stimulate regeneration after engraftment by promoting mitogenesis, chemotaxis and homing of endogenous stem cells [21, 22]. To our knowledge, professor Woodruff’s group was the first to evaluate the application of this type of scaffold for ovarian tissues, and was able to develop a functional bioengineered ovary based on the construct’s ability to produce hormones and initiate puberty in a mouse model [23]. Yet, only a few additional published studies using decellularized tissue as ovarian scaffolds have been reported since [24–28]. Collectively, these studies showed encouraging results when ECM-based materials were used as a supporting structure for ovarian cells, including the use of matrigel- and fibrin based scaffolds [29].

In an earlier study, we developed three different mouse ovarian scaffolds by decellularization [30]. Even if the mouse has a considerably different ovarian tissue composition and follicular growth compared with humans and other large mammals, we used this animal model so that potential differences between decellularization protocols may be discriminated, and challenging technical procedures for ovarian bioengineering applications can be optimized without the use of precious human material. Our previous study concluded that whole mouse ovaries can be decellularized in 10 h with a 0.5% sodium dodecyl sulfate (SDS) solution, or with a 2% sodium deoxycholate (SDC) solution for 16 h. We also evaluated the effects of a combination of these two detergents, but with a reduced exposure time, with the aim of preserving the ECM better while still effectively removing donor DNA. We concluded that there were some morphological and biological benefits of using the milder SDC over the more aggressive SDS. In the context of uterus bioengineering, these differences have been found to affect the functionality of the bioengineered tissue after engraftment [31, 32]. Furthermore, detergent-dependent differences in recellularization was reported during blood vessel reconstruction [33]. Hence, it is important to characterize any effects from the ovarian scaffold generation process in order to develop the best possible scaffold design. For these reasons, the goal of the current study was to evaluate in greater detail the ovarian scaffold structure and the remaining biological content after application of our three previously reported decellularization protocols for whole mouse ovaries [30]. Additionally, we quantified the recellularization efficiency and assessed the ability of the scaffolds to support mesenchymal stem cell (MSC) recellularization. Ovarian scaffolds that support MSCs may have a significant advantage since it has been shown that stroma cells support granulosa cells and estrogen production [34], can prevent follicle loss when added to grafted ovarian tissue [35], and have a therapeutic effect on follicle rejuvenation [36].

## Materials and methods

### Scaffold generation from isolated mouse ovaries

Oophorectomy was conducted on 108 female C57BL/6 N mice (Charles River, Sulzfeld, Germany) aged 10- to 20-weeks and resulted in 216 ovaries that were used for the analysis. The animal work was approved by the local animal ethics committee at the University of Gothenburg, Sweden (#114–2014). The isolated ovaries were dissected free from the surrounding tissue and immediately placed in Perfadex (Ex-vivo, Gothenburg, Sweden) and stored at − 20 °C before further processing.

For the decellularization experiments, the ovaries were thawed and randomly divided into three groups that were exposed to one of the three decellularization protocols previously reported [30]. In brief, the ovaries were submerged and agitated in 0.5% SDS for 10 h (protocol 1, P1; *n* = 63), or 2% SDC for 16 h (protocol 2, P2; *n* = 63), or a combination of the two detergents (0.5% SDS for 5 h followed by 2% SDC for 8 h; protocol 3, P3; *n* = 63). All the ovaries were then washed thoroughly in water, then treated for 30 min with a DNase I solution at 37 °C (40 units/ml; Sigma-Aldrich, Stockholm, Sweden). Each ovary was then washed with water for 24 h, then sterilized with 0.1% peracetic acid for 30 min, and washed for 24 h with sterile PBS supplemented with Gibco’s 1% antibiotic-antimycotic (Anti-Anti; penicillin 10,000 U/mL, streptomycin 10,000 μg/mL and fungizone 25 μg/mL; Thermo Fischer Scientific, Gothenburg, Sweden). All the ovaries were then frozen until analysis. A fourth group of normal unprocessed mouse ovaries that had been frozen once in Perfadex (Ex-vivo) was included as a normal control group for the analysis (*n* = 27).

### Histological analysis and DNA staining

All tissues that were processed for histology and immunohistochemistry were fixed in formaldehyde for 1 h, then dehydrated in alcohol and embedded in paraffin. Each ovarian tissue was then cut in a microtome into 5 μm sections. Selected midsections of each specimen were fixed on glass slides. Sections were then dewaxed and rehydrated prior to any staining procedure. Hematoxylin and eosin (H&E) staining was performed according to standard procedures. Some sections were processed with the nuclear dye 4′,6-diamidino-2-phenylindole (DAPI) for 1 min for fluorescence labelling of DNA. Excess DAPI was removed by two PBS washes, then slides were coverslipped with mounting media (F6182, Sigma Aldrich, Stockholm, Sweden). Stained sections were visualized and photographed with a Leica microscope (Micromedic AB, Stockholm, Sweden). To provide contrast for visualization of the scaffold in the images with DAPI staining, the auto fluorescence in the green channel was also photographed and included in the pictures.

### Quantification of collagen, glycosaminoglycans and elastin

Quantitative assessments of collagen, glycosaminoglycans (GAGs), and elastin were conducted using the kits from BioColor according to the manufacturer’s instructions (Fastin™ Elastin Assay; Blyscan™ Sulfated Glycosaminglycan Assay; Sircol™ Soluble Collagen Assay; Sircol™ Insoluble Collagen; Carrickfergus, Northern Ireland, UK). Due to the detection limitations of the kits (5 μg for elastin; 0.25 μg for GAGs, 1.0 μg for soluble collagen, and 10.0 μg for insoluble collagen), two ovary samples were pooled for each of the analysis. Therefore, the results are presented as “μg ECM component/two ovaries”. A total of six normal ovaries were used for the analysis of each component, and a total of 12 decellularized ovaries were used per group and per analysis.

### Evaluation of scaffold toxicity

Scaffolds generated from decellularized tissues may be toxic if remnants of the detergents are present due to insufficient washing. Therefore, a toxicity test was conducted using a 3-(4,5-dimethylthiazol-2-yl)-2,5-diphenyltetrazolium bromide (MTT) assay, which indirectly measures the bioactivity of cells based on the ability of NAD(P)H-dependent cellular oxidoreductase to convert the yellow MTT dye to the stable purple dye called formazan. The reduction of MTT to formazan is quantified based on the detected absorbance in the wavelengths between 500 and 600 nm (a high formazan conversion rate indicate a healthy cell metabolism). The analysis was done using Roche’s Cell Proliferation Kit 1 (MTT; Merck KGaA, Darmstadt, Germany) according to their instructions. Briefly, 5000 human embryonic kidney cells (HEK293) were cultured in standard conditions in a 96-well plate in 100 μl cell culture medium (Dulbecco’s modified eagle medium (DMEM), supplemented with Glutamax, 10% fetal calf serum (FCS), and 1% Anti-Anti (all were Gibco products from Thermo Fischer Scientific). Some wells that contained media without any cells served later as “the blank”. Half of the medium was replaced with liquid from the final wash during the decellularization process (P1-P3; *n* = 4 per group). A solution containing 20% SDS was used for cell death-control, and fresh medium was used as the non-toxic live-cell control. After an 8 h incubation period, 10 μl of MTT dye was added to each culture well and the cells were further incubated overnight. Purple formazan crystals converted from the MTT dye by metabolically active cells were measured in a plate reader at 565 nm.

### Recellularization with red fluorescent protein labelled MSCs and fluorescent staining

To evaluate whether the produced ovarian scaffolds were sterile, non-cytotoxic, and were capable of harboring cells that can be of therapeutic benefit for follicular support, red fluorescent protein (RFP)-labelled mouse bone marrow MSCs (RFP-MSCs; MUBMX-01201 C57BL/6, Cyagen, Santa Clara, CA, USA) were cultured up to the seventh passage under standard culture conditions (DMEM with Glutamax, 10% fetal bovine serum, 1% Antibiotic-Antimycotic, Thermo Fisher Scientific; humidified chamber with 5% CO_2_ enriched air). For ovarian scaffold recellularization, one million cells were used to recellularize each ovarian scaffold by five repeated injections of 200,000 cells per injection. Injections were placed to cover the entire structure of the ovary using a 30G needle connected to a 1 ml syringe (VWR International, Stockholm, Sweden). Each recellularized ovarian construct (*n* = 12 per scaffold group) was then left for 30 min in the incubator to allow for cell attachment. Following this, additional cell culture medium was added and the constructs were incubated individually in a 6-well plate for 14 days. The culture medium was replaced every second or third day depending on the pH-sensitive color change in the culture medium. Nine constructs from each group were then washed in PBS, fixed with paraformaldehyde and embedded in paraffin blocks and histologically processed as described above (for H&E staining). Immunohistochemistry for RFP was also conducted on sections of recellularized ovarian scaffolds. This was necessary since the paraffin embedment negated the biologically active RFP that was expressed by the genetically engineered cells. For antibody staining, antigen retrieval was performed with citrate buffer (pH = 6.0) in a pressure cooker and 10% normal goat serum diluted in PBS containing 0.2% triton-X100 was used for blocking (1 h). The sections were then incubated with the following antibodies for 1 h at room temperature; RFP (1:100; #ab62341, Abcam, Cambridge, UK), cleaved caspase-3 (1:100; asp175, Cell signaling, Leiden, The Netherlands) and Ki67 (1:100; #16667, Abcam). After several washes in PBS, the fluorescent-labelled secondary antibodies Alexa Fluor 488 (1:300; #150077, Abcam) and/or Alexa Fluor 594 (1:300; #150116, Abcam) were added to the sections for 1 h at room temperature. Sections from a processed mouse spleen was used for a positive control for the staining of Ki67 and cleaved caspase-3 (data not shown).

The recellularization efficiency was manually assessed by observation of the scanned H&E stained 5 μm sections. Cells that were within, and on the surface of the ovarian scaffolding structure were counted. Cells located outside the actual scaffolding structure were not counted. The total counting area was measured and the total number of cells per square millimeter was calculated.

### Scanning electron microscopy

Normal, decellularized and recellularized mouse ovaries (*n* = 3 per group) were processed for scanning electron microscopy (SEM). Due to the absence of cellular lipid membranes in the decellularized tissues, better contrast was obtained in the specimen for SEM after they were treated with osmium tetroxide and thiocarbohydrazide [37]. The treated samples were dehydrated, mounted, sputter coated with 4 nm gold particles and imaged with a Zeiss Gemini-2 SEM 500 (Carl Zeiss AB, Stockholm, Sweden).

### Statistical analysis

All the data were found to be non-parametric according to the Shapiro-Wilk test. Therefore, the Kruskal-Wallis test and Dunn’s multiple group comparison were used to evaluate any significant differences. In some cases, when only two groups were compared, the Mann-Whitney U-test was conducted. Data are presented as box plots with medians and the respective interquartile range (10th to 90th percentile). Differences were considered significant at *p* < 0.05.

## Results

### Decellularization, quantification of ECM components and cytotoxicity test

**Fig. 1:**
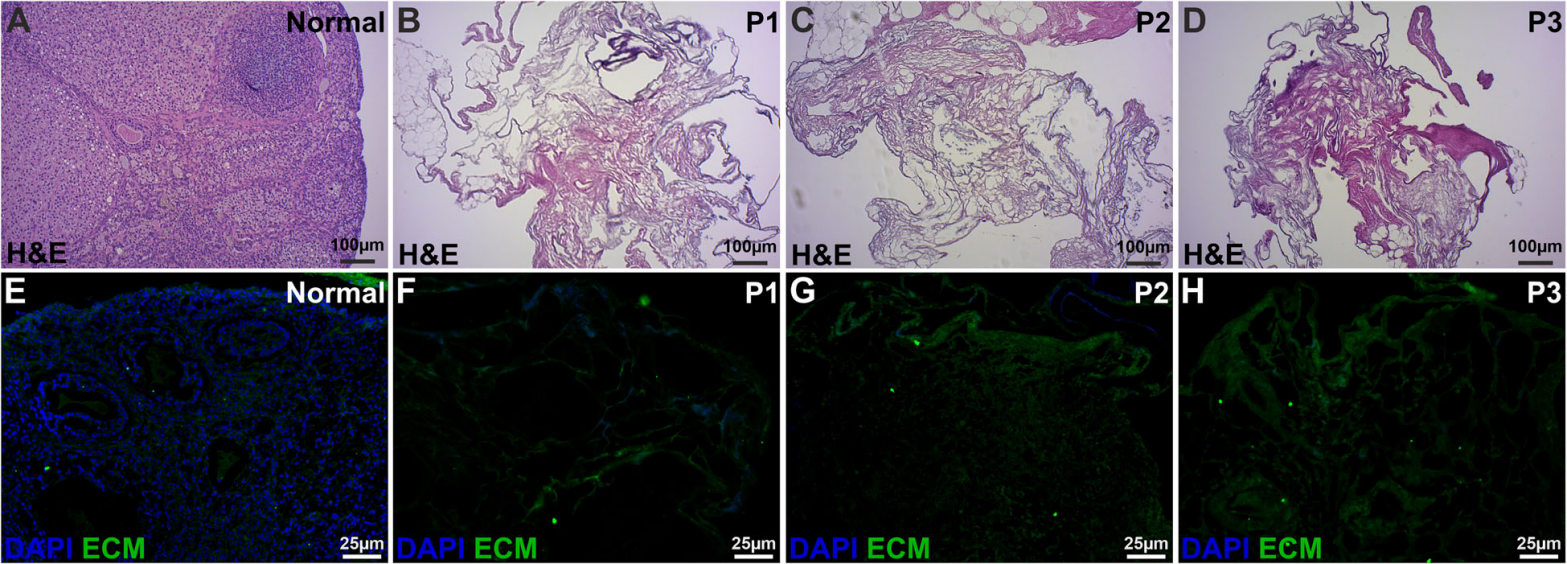
Staining analysis of decellularized mouse ovary scaffolds. Decellularization was confirmed by two different staining methods; hematoxylin and eosin (H&E; **a-d**) and with the DNA-labelling dye DAPI (**e-h**). No dark-stained nuclei could be detected in the decellularized ovaries in any of the produced scaffolds (**a-d**). DAPI is considered to be a more than H&E and also confirmed the removal of DNA after decellularization (**e-h**). The green stain represents auto fluorescence from the ovarian tissue and was included in the images to enable the visualization of the extracellular matrix (ECM) structure

All three decellularization methods were found to be effective based on the successful removal of intracellular components and nuclear material. The ovary-specific extracellular scaffolding structure was better preserved with P1 and P2, than with P3 (Fig. 1).

**Fig. 2:**
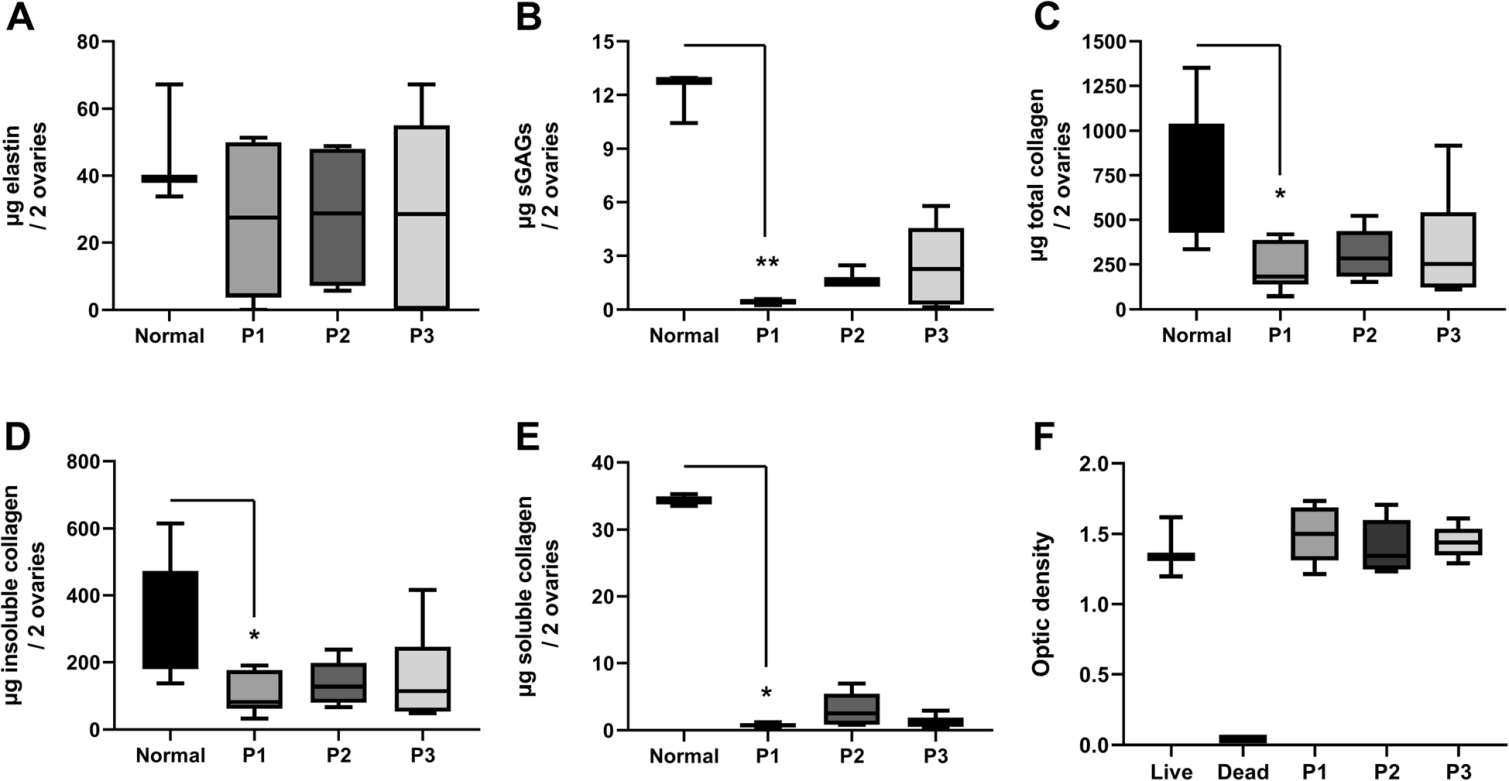
The levels of the important extracellular matrix components elastin (**a**), sulfated glucosaminoglycans (sGAGs; **b**) and collagen (total, insoluble- and soluble collagen, respectively; **c-e**) were quantified before and after decellularization with the various protocols (P1, P2, and P3 respectively). An MTT toxicity test was also conducted to confirm the complete removal of toxic remnants from the decellularization process which indicated that the scaffolds were non-toxic (F)

The mean elastin content in the decellularized ovarian tissue was about 60% compared with the original ovarian elastin content prior to decellularization. This reduction was not significant compared with normal control tissue due to large variations between samples in each group. Protocol 1-treatment lead to a significant decrease of sGAGs, and the levels of both insoluble and soluble collagens were significantly reduced by P1 based on multiple group comparison statistics (Kruskal-Wallis test and Dunn’s multiple group comparison) (Fig. 2a-e). When each of the test groups was compared with the normal group by the Mann-Whitney U-test, P2 and P3 were found to significantly reduce the levels of sGAGs and collagen (*p* < 0.05 vs. the normal group). However, the levels of sGAGs, and to some degree also the soluble collagen levels, were higher after P2 than after P1 when these groups were compared with each other using the Mann-Whitney U-test (sGAGs: *p* = 0.0022, soluble collagen: *p* = 0.065).

Finally, the MTT-assay indicated that no cytotoxic remnants were present in any of the three scaffold types (Fig. 2f).

### Recellularization efficiency based on staining and scanning electron microscopy

**Fig. 3:**
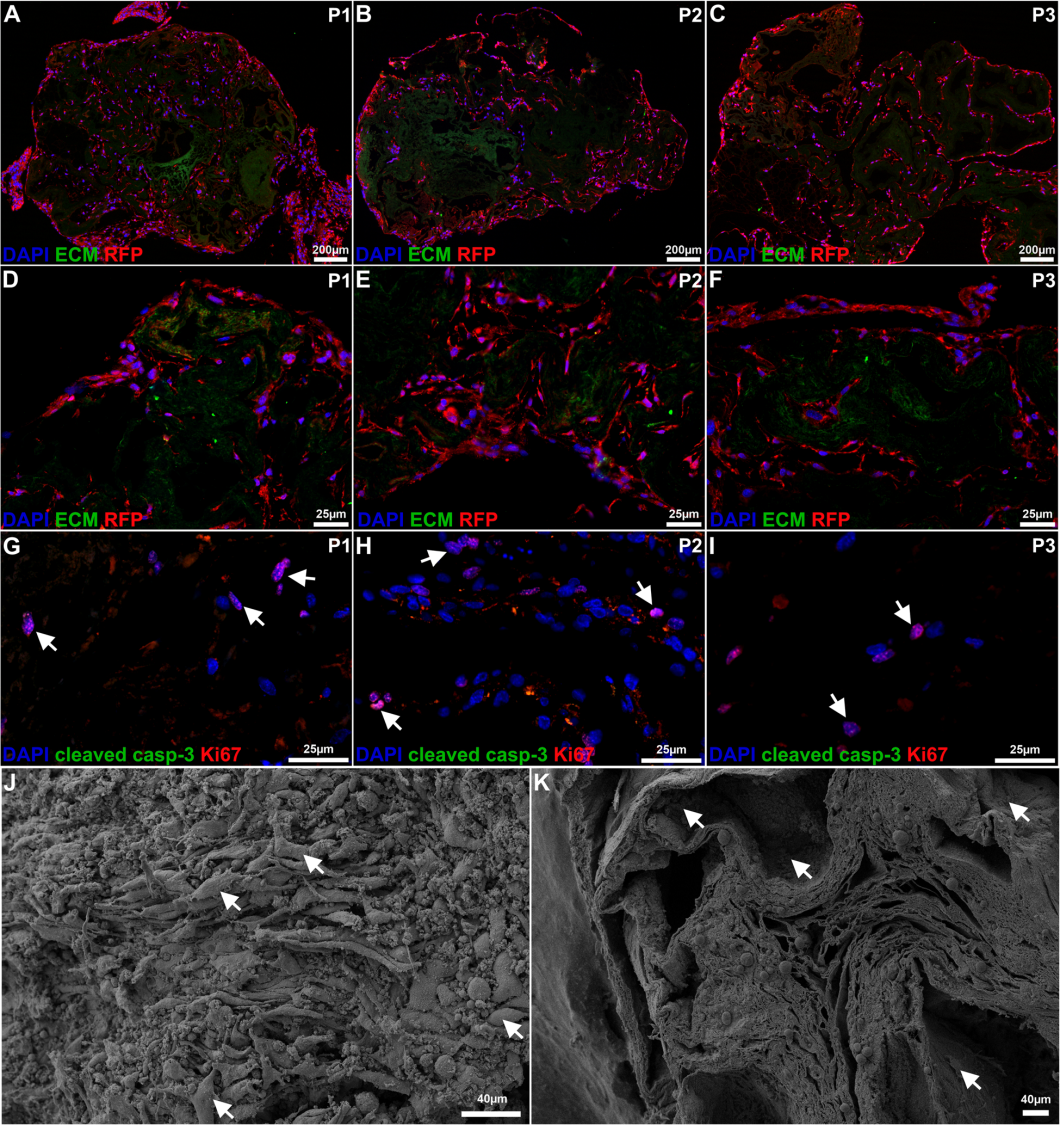
Evaluation of recellularization and biocompatibility of the decellularized scaffolds. Red fluorescent protein (RFP)-labelled mesenchymal stem cells were used for recellularization. The cells recolonized large areas of the ovarian scaffolds, including the deeper layers of all scaffold types. However, the cortical area of the scaffolds accommodated more cells than the medulla regions (low power magnification **a-c**; high magnification (**d-i**). All the detected cells were double-labeled with RFP (red) and DAPI (blue; **a-f**), and indicated that all cells originated from the recellularization episode and were not the result of an incomplete decellularization. Many cells were positive for the proliferation marker Ki67 (red, arrows point out examples of positive cells), while the cells remained negative for the apoptotic marker cleaved caspase-3 (green; **g-i**) after 2 weeks in vitro. The surface of the scaffolds was densely populated with cells in all scaffold types, as confirmed by both immunocytochemistry (**a-i**) and scanning electron microscopy (**j-k**; arrows indicate the cell structures in a recellularized construct after the application of the decellularization protocol 1 (P1). It was more difficult to visualize cells in cross sectioned recellularized scaffolds with SEM. However, cells that were morphologically unaffected by the sampling preparation could be visualized in the hollow- and porous structures of the ovarian scaffolds, including the deeper scaffold compartments (**k**; the arrows indicate cell structures). Green color (**a-f**) represents auto fluorescence from the ovarian tissue and was included in the images to enable the visualization of the extracellular matrix (ECM) structure

The RFP-labelled MSCs that were used for the recellularization were able to recolonize large areas of the ovarian scaffolds, including the deeper layers (Fig. 3a-f). All the detected cells were double-labeled with RFP and DAPI, demonstrating that they were all recellularized cells, and did not originate from the scaffold donor as a result of an incomplete decellularization. In general, the ovarian cortex exhibited better recellularization than the medulla-region in all scaffold types. Sections stained with Ki67 and cleaved caspase-3 showed that cells within the scaffolds remained proliferative and non-apoptotic in all constructs 14 days after cell seeding (Fig. 3g-i).

Highly detailed topography assessments of all three types of recellularized ovarian scaffolds with SEM showed that the remaining ovarian ECM structure was significantly repopulated on the surface (Fig. 3j, arrows). It was difficult to visualize the cells in cross sectioned recellularized scaffolds with SEM. However, cells were visualized in the hollow and porous structures, including in the deeper scaffold compartments (Fig. 3k, arrows).

**Fig. 4:**
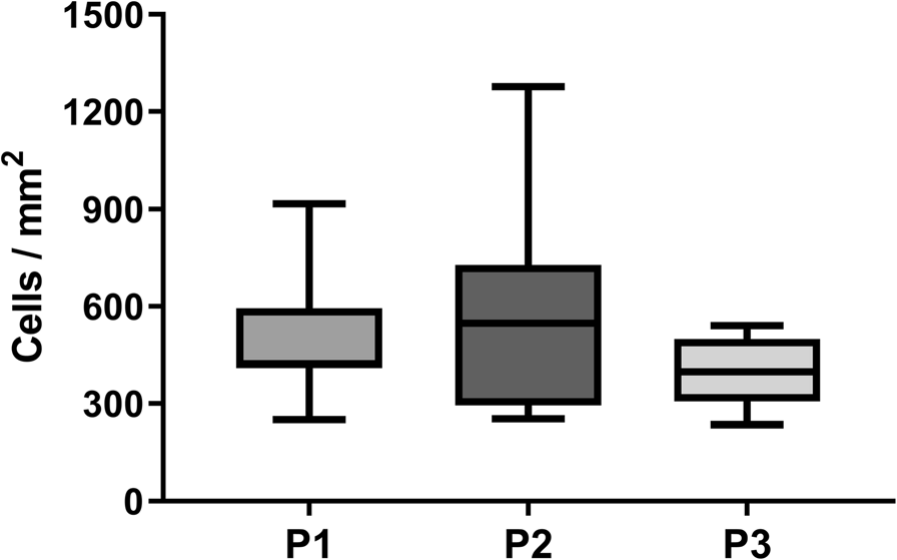
Cell density measurement 2 weeks after recellularization. Cell density was quantified in cross sectioned ovarian constructs after 2 weeks in vitro. There was no significant difference between the three recellularized ovarian scaffold types with regard to the number of cells per mm^2^. However, there was a large intra-group variance, particularly among P2-derived constructs, several of which displayed extensive recolonization

The cell density data indicated that there was no significant difference in cell density between the three recellularized ovarian scaffold types (Fig. 4). However, there was a large intra-group variance (evident by the large error bars). For example, one ovarian scaffold from the P2-treated group was extensively recellularized and had an average of 1277 cells/mm^2^, which is a considerable difference compared with the average number for the most successful construct in the P1-derived scaffolds (916 cells/mm^2^) and the P3-derived scaffolds (541 cells/mm^2^; Fig. 4). However, when comparing the cell density of the least efficiently recellularized scaffold in each group, no major difference was seen (236–253 cells/mm^2^ for all the three groups). Hence, even if P2 had several more constructs with greater recellularization efficiency than scaffolds produced by P1 or P3, we did not observe a significant difference between the construct groups due to the inconsistency of recellularization efficiency within each of the groups.

## Discussion

Decellularized organs have a tissue-specific ECM arrangement with growth factors and a microenvironment that cells can modify after reconstruction [38, 39]. This is a critical merit for an ovarian biomaterial. Therefore, a number of bioengineering studies investigated decellularized ovarian tissue from both small and large animals, including humans [23–26, 28, 40]. Most of these studies used SDS as the detergent for scaffold generation. However, due to chemical differences of various detergents, altered decellularization protocols generate different scaffolding types with unique physical- and biological properties that may impact the construct quality and downstream applications [32, 33]. SDS is considered an aggressive ionic detergent that denatures proteins by breaking non-covalent bonds, including hydrogen bonds, hydrophobic and ionic protein interactions that are responsible for the native 3D protein structure. It has also been known to reduce growth factor levels, sGAGs and collagens, and limit the recellularization efficiency [41]. Sodium deoxycholate is another ionic detergent, but is considerably milder than SDS. Its predominant decellularization action involves cell membrane disruption, while preserving the structural proteins better than SDS [42]. The detergent concentration used for decellularization is also an important variable for the outcome of scaffold function. Human ovarian cortex and medulla segments similar in size to whole mouse ovaries were successfully decellularized and recellularized after a 18 h–24 h SDS protocol with a lower concentration (0.1%) than used in the present study [28]. The results of the latter study showed that their protocol formed a well preserved ECM that could support early stage follicular growth. Alternatively, another group used 1% SDS to decellularize whole mouse ovaries (3 h), and sheep- and human ovarian tissue strips (overnight treatment), and compared the produced scaffolds with a sodium hydroxide-based decellularization protocol which they found better than their SDS-protocol [40]. Given all these conflicting and varying findings, it is important to continue to investigate scaffold generation protocols and the associated bioengineering techniques. Rodent studies are undoubtedly beneficial for this type of work as the material is more easily accessible and more rapidly available than large animal tissue or human material.

We assessed three decellularization strategies for scaffolding generation in an earlier study which morphologically indicated that SDC may be more advantageous than SDS [30]. In this follow-up study, we conducted a much more detailed assessment of the remaining ECM after application of the same decellularization protocols. These new results show that there was a greater loss of water-soluble sGAGs and collagens with the SDS protocol than with the SDC protocol. Although the loss of these important ECM molecules may be compensated for by priming the scaffolds with respective molecule prior to recellularization, it may still negatively impact on the repopulation of the ovarian scaffolds. For these reasons, we also evaluated the bioactivity and cell-supporting properties of the ovarian scaffolds. The cytotoxicity test confirmed the absence of potential toxic remnants from the decellularization process. Further, RFP-labelled MSCs that were used for recellularization showed remarkably good cell distribution 2 weeks after the cell injections. Thus, our recellularization results suggest; a) good technical application of the RFP-labelled cells, b) that they were well supported by the scaffolds, and c) that 2 weeks may be enough time to culture recellularized mouse ovarian constructs to ensure widespread MSC distribution. The results further demonstrate that growth medium diffusion through the scaffold is sufficient to maintain cells alive, even in the deeper compartments of the scaffolds. These are significant results, since limited cell distribution following recellularization of decellularized tissue has consistently been shown to be one of the biggest hurdles when using decellularized tissue for bioengineering studies [43]. Interestingly, when different cell quantities were evaluated for the recellularization of decellularized sheep uterus tissue slices, the use of a higher cell number at initiation did not have any recellularization advantages when analyzed 14 days later [44]. For these reasons, we did not compare recellularization efficiency with different cell quantities in this study. However, it has been reported that longer in vitro cultures could increase cell density further [28], and strategies that facilitate culture media circulation during the process could be advantageous [45].

The staining for Ki67 (proliferation) and cleaved caspase-3 (apoptosis) suggest that our cells in the ovarian scaffolds were still proliferating after 14 days, and that the recellularization efficiency may have increased if the constructs remained in culture for an extended time period.

Continued reports on how to improve recellularization are of great significance for future success using decellularized tissue for ovarian bioengineering applications, including how to best apply the cells and the follicles to the scaffolds. It is our opinion that labelled cells should be used for this type of studies in order to clearly distinguish between the newly added cells and any remnant donor cells that may linger from an incomplete decellularization process. It could otherwise lead to overestimation of the true recellularization efficiency. Therefore, we used commercially available RFP-labelled MSCs in this study. Our findings clearly showed that all cells in the scaffold were RFP^+^, and thus, originated from the recellularization phase. However, we noticed a widespread intra-group difference in the cell density of our constructs. Thus, there might be some technical-related variability in the recellularization process. This problem was also highlighted by Pors et al. (2019) and the common finding further underscores the value of using small animal models to standardize and optimize protocols for the recellularization process.

We think that including stroma cells (such as MSCs) can be advantageous for ovarian bioengineering applications since they support granulosa cells and estrogen production [34] and have follicle-rejuvenating effects when transplanted into the ovary [36, 46]. Scanning electron microscopy images of our recellularized constructs visualized a MSC-dense surface that may encourage revascularization, provide protection from graft degeneration, and stimulate intrinsic regenerative responses following engraftment [32, 47]. We therefore speculate that inclusion of these cells with follicles in future studies may be advantageous for the bioengineered ovarian constructs. The next goal of our research will be to assess whether murine and/or human ovarian follicles can be supported in these bioengineered constructs in vitro, and/or in vivo by e.g. using xenograft animal models, and assess the constructs ability to support hormone production and oocyte maturation.

In conclusion, our SDC protocol seemed slightly more advantageous compared with our two SDS protocols (P1 and P3). However, all scaffold types were found biocompatible, based on the recellularization efficiency obtained after 2 weeks in vitro with MSCs. The MCSs were widely distributed in all compartments of the scaffolds, but showed a more profound density in the cortex region. The recellularization efficiency was particularly high in several SDC-produced (P2) scaffolds which further suggests that this decellularization detergent may be advantageous for ovarian scaffold generation.

## Data Availability

The protocols described in the manuscript, or any relevant raw data, will be freely available on request to any scientist wishing to use them for non-commercial purposes.

## Abbreviations

3D: Three-dimensional
AB: Alcian blue
Anti-Anti: Gibco’s antibiotic-antimycotic
DAPI: 4′,6-diamidino-2-phenylindole
ECM: Extracellular matrix
H&E: Hematoxylin and eosin
MSC: Mesenchymal stem cell
MTT: 3-(4,5-dimethylthiazol-2-yl)-2,5-diphenyltetrazolium bromide
PBS: Phosphate buffered saline
RFP: Red fluorescent protein
RFP-MSCs: Red fluorescent protein labelled mouse bone marrow mesenchymal stem cells
SDC: Sodium deoxycholate
SDS: Sodium dodecyl sulfate
SEM: Scanning electron microscopesGAG - sulfated glycosaminoglycan

## References

[1] Pereira N, Schattman GL (2017). Fertility preservation and sexual health after Cancer therapy. J Oncol Pract.

[2] Wallace WH, Thomson AB, Kelsey TW (2003). The radiosensitivity of the human oocyte. Hum Reprod.

[3] Diaz-Garcia C, Domingo J, Garcia-Velasco JA, Herraiz S, Mirabet V, Iniesta I, Cobo A, Remohi J, Pellicer A. Oocyte vitrification versus ovarian cortex transplantation in fertility preservation for adult women undergoing gonadotoxic treatments: a prospective cohort study. Fertil Steril. 2018. 10.1016/j.fertnstert.2017.11.018 29428307

[4] Moravek MB, Confino R, Smith KN, Kazer RR, Klock SC, Lawson AK, Gradishar WJ, Pavone ME (2018). Long-term outcomes in cancer patients who did or did not pursue fertility preservation. Fertil Steril.

[5] Donnez J, Dolmans MM, Pellicer A, Diaz-Garcia C, Sanchez Serrano M, Schmidt KT, Ernst E, Luyckx V, Andersen CY (2013). Restoration of ovarian activity and pregnancy after transplantation of cryopreserved ovarian tissue: a review of 60 cases of reimplantation. Fertil Steril.

[6] Ribeiro R, Rebolho JC, Tsumanuma FK, Brandalize GG, Trippia CH, Saab KA (2017). Uterine transposition: technique and a case report. Fertil Steril.

[7] Donnez J, Dolmans MM (2017). Fertility preservation in women. N Engl J Med.

[8] Dolmans MM, Marinescu C, Saussoy P, Van Langendonckt A, Amorim C, Donnez J (2010). Reimplantation of cryopreserved ovarian tissue from patients with acute lymphoblastic leukemia is potentially unsafe. Blood.

[9] Chiti MC, Dolmans MM, Donnez J, Amorim CA. Fibrin in reproductive tissue engineering: a review on its application as a biomaterial for fertility preservation. Ann Biomed Eng. 2017. 10.1007/s10439-017-1817-5 28271306

[10] Fisch B, Abir R (2018). Female fertility preservation: past, present and future. Reproduction.

[11] McLaughlin M, Albertini DF, Wallace WHB, Anderson RA, Telfer EE (2018). Metaphase II oocytes from human unilaminar follicles grown in a multi-step culture system. Mol Hum Reprod.

[12] Luyckx V, Dolmans MM, Vanacker J, Scalercio SR, Donnez J, Amorim CA (2013). First step in developing a 3D biodegradable fibrin scaffold for an artificial ovary. J Ovarian Res.

[13] Vanacker J, Dolmans MM, Luyckx V, Donnez J, Amorim CA (2014). First transplantation of isolated murine follicles in alginate. Regen Med.

[14] Xu M, Kreeger PK, Shea LD, Woodruff TK (2006). Tissue-engineered follicles produce live, fertile offspring. Tissue Eng.

[15] Shikanov A, Smith RM, Xu M, Woodruff TK, Shea LD (2011). Hydrogel network design using multifunctional macromers to coordinate tissue maturation in ovarian follicle culture. Biomaterials.

[16] Laronda MM, Rutz AL, Xiao S, Whelan KA, Duncan FE, Roth EW, Woodruff TK, Shah RN (2017). A bioprosthetic ovary created using 3D printed microporous scaffolds restores ovarian function in sterilized mice. Nat Commun.

[17] Shea LD, Woodruff TK, Shikanov A (2014). Bioengineering the ovarian follicle microenvironment. Annu Rev Biomed Eng.

[18] Xiao S, Zhang J, Romero MM, Smith KN, Shea LD, Woodruff TK (2015). In vitro follicle growth supports human oocyte meiotic maturation. Sci Rep.

[19] Campo H, Cervello I, Simon C (2017). Bioengineering the uterus: an overview of recent advances and future perspectives in reproductive medicine. Ann Biomed Eng.

[20] Hellström M, Bandstein S, Brännström M (2017). Uterine tissue engineering and the future of uterus transplantation. Ann Biomed Eng.

[21] Peloso A, Dhal A, Zambon JP, Li P, Orlando G, Atala A, Soker S (2015). Current achievements and future perspectives in whole-organ bioengineering. Stem Cell Res Ther.

[22] Reing JE, Zhang L, Myers-Irvin J, Cordero KE, Freytes DO, Heber-Katz E, Bedelbaeva K, McIntosh D, Dewilde A, Braunhut SJ, Badylak SF (2009). Degradation products of extracellular matrix affect cell migration and proliferation. Tissue Eng Part A.

[23] Laronda MM, Jakus AE, Whelan KA, Wertheim JA, Shah RN, Woodruff TK (2015). Initiation of puberty in mice following decellularized ovary transplant. Biomaterials.

[24] Liu WY, Lin SG, Zhuo RY, Xie YY, Pan W, Lin XF, Shen FX (2017). Xenogeneic Decellularized scaffold: a novel platform for ovary regeneration. Tissue Eng Part C Methods.

[25] Hassanpour A, Talaei-Khozani T, Kargar-Abarghouei E, Razban V, Vojdani Z (2018). Decellularized human ovarian scaffold based on a sodium lauryl ester sulfate (SLES)-treated protocol, as a natural three-dimensional scaffold for construction of bioengineered ovaries. Stem Cell Res Ther.

[26] Jakus AE, Laronda MM, Rashedi AS, Robinson CM, Lee C, Jordan SW, Orwig KE, Woodruff TK, Shah RN. "tissue papers" from organ-specific Decellularized extracellular matrices. Adv Funct Mater. 2017;27. 10.1002/adfm.201700992 PMC5665058 29104526

[27] Oktay K, Bedoschi G, Pacheco F, Turan V, Emirdar V (2016). First pregnancies, live birth, and in vitro fertilization outcomes after transplantation of frozen-banked ovarian tissue with a human extracellular matrix scaffold using robot-assisted minimally invasive surgery. Am J Obstet Gynecol.

[28] Pors SE, Ramlose M, Nikiforov D, Lundsgaard K, Cheng J, Andersen CY, Kristensen SG (2019). Initial steps in reconstruction of the human ovary: survival of pre-antral stage follicles in a decellularized human ovarian scaffold. Hum Reprod.

[29] Sadr SZ, Fatehi R, Maroufizadeh S, Amorim CA, Ebrahimi B. Utilizing fibrin-alginate and Matrigel-alginate for mouse follicle development in three-dimensional culture systems. Biopreserv Biobank. 2018. 10.1089/bio.2017.0087 29363997

[30] Alshaikh AB, Padma AM, Dehlin M, Akouri R, Song MJ, Brannstrom M, Hellstrom M (2019). Decellularization of the mouse ovary: comparison of different scaffold generation protocols for future ovarian bioengineering. J Ovarian Res.

[31] Hellström M, El-Akouri RR, Sihlbom C, Olsson BM, Lengqvist J, Bäckdahl H, Johansson BR, Olausson M, Sumitran-Holgersson S, Brännström M (2014). Towards the development of a bioengineered uterus: comparison of different protocols for rat uterus decellularization. Acta Biomater.

[32] Hellström M, Moreno-Moya JM, Bandstein S, Bom E, Akouri RR, Miyazaki K, Maruyama T, Brännström M (2016). Bioengineered uterine tissue supports pregnancy in a rat model. Fertil Steril.

[33] Simsa R, Padma AM, Heher P, Hellstrom M, Teuschl A, Jenndahl L, Bergh N, Fogelstrand P (2018). Systematic in vitro comparison of decellularization protocols for blood vessels. PLoS One.

[34] Sittadjody S, Enck KM, Wells A, Yoo JJ, Atala A, Saul JM, Opara EC. Encapsulation of Mesenchymal stem cells in 3D ovarian cell constructs promotes stable and long-term hormone secretion with improved physiological outcomes in a syngeneic rat model. Ann Biomed Eng. 2019. 10.1007/s10439-019-02334-w PMC7021574 31367915

[35] Xia X, Yin T, Yan J, Yan L, Jin C, Lu C, Wang T, Zhu X, Zhi X, Wang J (2015). Mesenchymal stem cells enhance angiogenesis and follicle survival in human cryopreserved ovarian cortex transplantation. Cell Transplant.

[36] Herraiz S, Romeu M, Buigues A, Martinez S, Diaz-Garcia C, Gomez-Segui I, Martinez J, Pellicer N, Pellicer A (2018). Autologous stem cell ovarian transplantation to increase reproductive potential in patients who are poor responders. Fertil Steril.

[37] Davies S, Forge A (1987). Preparation of the mammalian organ of Corti for scanning electron microscopy. J Microsc.

[38] Hillebrandt KH, Everwien H, Haep N, Keshi E, Pratschke J, Sauer IM (2019). Strategies based on organ decellularization and recellularization. Transpl Int.

[39] Caralt M, Uzarski JS, Iacob S, Obergfell KP, Berg N, Bijonowski BM, Kiefer KM, Ward HH, Wandinger-Ness A, Miller WM (2015). Optimization and critical evaluation of decellularization strategies to develop renal extracellular matrix scaffolds as biological templates for organ engineering and transplantation. Am J Transplant.

[40] Eivazkhani F, Abtahi NS, Tavana S, Mirzaeian L, Abedi F, Ebrahimi B, Montazeri L, Valojerdi MR, Fathi R (2019). Evaluating two ovarian decellularization methods in three species. Mater Sci Eng C Mater Biol Appl.

[41] Paulo Zambon J, Atala A, Yoo JJ (2019). Methods to generate tissue-derived constructs for regenerative medicine applications. Methods.

[42] Gilpin A, Yang Y (2017). Decellularization strategies for regenerative medicine: from processing techniques to applications. Biomed Res Int.

[43] Rana D, Zreiqat H, Benkirane-Jessel N, Ramakrishna S, Ramalingam M (2017). Development of decellularized scaffolds for stem cell-driven tissue engineering. J Tissue Eng Regen Med.

[44] Tiemann TT, Padma AM, Sehic E, Backdahl H, Oltean M, Song MJ, Brannstrom M, Hellstrom M. Towards uterus tissue engineering: a comparative study of sheep uterus decellularisation. Mol Hum Reprod. 2020. 10.1093/molehr/gaaa009 PMC7103571 31980817

[45] Mirzaeian L, Eivazkhani F, Hezavehei M, Moini A, Esfandiari F, Valojerdi MR, Fathi R (2020). Optimizing the cell seeding protocol to human Decellularized ovarian scaffold: application of dynamic system for bio-engineering. Cell J.

[46] Lai D, Wang F, Yao X, Zhang Q, Wu X, Xiang C (2015). Human endometrial mesenchymal stem cells restore ovarian function through improving the renewal of germline stem cells in a mouse model of premature ovarian failure. J Transl Med.

[47] Dorin RP, Pohl HG, De Filippo RE, Yoo JJ, Atala A (2008). Tubularized urethral replacement with unseeded matrices: what is the maximum distance for normal tissue regeneration?. World J Urol.

